# Editorial: A multidisciplinary approach to treatment of severe chronic airway disease (CAD): focus on biomarkers

**DOI:** 10.3389/falgy.2024.1357886

**Published:** 2024-02-02

**Authors:** Raquel López-Rodríguez, Irina Bobolea, Carlos Melero, Manuel J. Rial

**Affiliations:** ^1^ Department of Allergy, Lucus Augusti University Hospital, Lugo, Spain; ^2^ Department of Allergy, Institut Clínic Respiratorio, Hospital Clinic of Barcelona, Barcelona, Spain; ^3^ Network Biomedical Research Center for Respiratory Diseases, Carlos III Health Institute (ISCIII), Madrid, Spain; ^4^ Department of Pulmonology, Research Institute Hospital 12 de Octubre, Madrid, Spain; ^5^ Department of Allergy, A Coruña University Hospital Complex (CHUAC), A Coruña, Spain

**Keywords:** asthma, monoclonal antibodies, severe asthma, biomarkers, FeNO (Fraction of exhaled Nitric Oxide)

**Editorial on the Research Topic** A multidisciplinary approach to treatment of severe chronic airway disease (CAD): focus on biomarkers

Severe asthma (SA) is a complex disease that requires a specialized and multidisciplinary approach (1). Its prevalence varies depending on the territory (20% in the USA-Western Europe and −30% in Central Europe) (2). It is estimated that half of these severe patients have poor control of their disease, although in global terms it corresponds to 5%–10% of the total asthmatic population (2). The best possible management requires a correct diagnosis, an adequate continuity of care and the choice of the best available treatment, thanks to the identification of the predominant phenotype with the help of clinical, biological, and pulmonary function markers (1–4). Peripheral blood eosinophilia, atopy, and the value of FENO, as biomarkers of T2 response, help with the indication of the monoclonal Antibodies (mAb) that target either T2 prototypic interleukins or their receptors (mepolizumab, reslizumab, benralizumab and dupilumab), or IgE (omalizumab) (2, 5). The so-called non-T2 phenotype is a catch-all that encompasses mechanisms as diverse as neutrophilic asthma derived from infections, or pauciinflammatory asthma, where the recently approved Tezepelumab (human anti-TSLP mAb) seems to acquire relevance. Tezepelumab has shown great benefits in the control and quality of life of the disease, across all T2 and non-T2 spectra (1, 2).

Monoclonal antibodies have been postulated as the most promising therapeutic option in the management of SA in the last decade (6). They are able to modify the disease-specific immune response and have been shown to be effective in reducing exacerbations, improving lung function and increasing quality of life in severe patients who remain uncontrolled to high-dose conventional treatments (1, 2). Although traditionally they have always been reserved for advanced stages of treatment, concerns arise as to whether these benefits could be extended to patients in earlier stages of the disease.

The main objective of asthma treatment is to achieve and maintain control of the disease as soon as possible, in addition to preventing exacerbations, chronic airflow obstruction and minimizing mortality and medication side effects, mainly systemic corticosteroids (1–3). Three months are recommended to assess the effects of inhaled therapy on symptoms and lung function, and a minimum of 4–6 months for a first assessment of clinical, functional, and inflammatory response in patients treated with mAb (2). In case of non-response or insufficient response, other alternatives also appear, allowing a change of biologic to be considered in certain situations, on the understanding that, even if the initial indication was correct, the response to treatment is never complete. Clinical remission is defined as 12 or more months without significant symptoms (measured by an appropriate and validated instrument such as asthma control test), optimization or stabilization of pulmonary function, and no use of systemic corticosteroids (1). In this Research Topic, Rial and Domínguez-Ortega propose to differentiate remission into three categories of qualitative response: clinical remission, inflammatory remission (objective resolution of inflammation is required) and complete remission (no symptoms, no inflammation, without medication).

Several potential causes of suboptimal response to monoclonals have been described: incorrect identification of aT2 endotype; heterogeneity of the T2 response (different pathways involved- overlap); concomitant diseases (obesity, gastroesophageal reflux, anxiety-depression, and even refractory nasal polyps) that can produce exacerbations even in patients receiving mAbs.

To avoid errors in phenotyping, Marcos and Cisneros Serrano in their article estimate that FeNO measurement can help in the phenotyping process of severe asthmatics, by detecting those patients who may benefit from biologic therapies targeting type 2 inflammation. Furthermore, by considering the heterogeneous nature of asthma, where different phenotypes and endotypes coexist, biologic treatments could be tailored in a more personalized and precise manner from the outset. López-Viña et al. in their article propose a therapeutic algorithm in severe asthma as a multifactorial process based on biomarkers, steroid dependence, and clinical comorbidities. This could lead to a better response to treatment, minimizing the need for constant adjustments and providing more consistent results.

Something that adds further complexity in the field of severe asthma are the changes in the inflammatory phenotype (which usually does not remain stable over time): it varies in about half of the patients, mainly due to external factors such as intercurrent respiratory infections or smoking, and less frequently to treatment aimed at reducing bronchial or blood eosinophilia (1). In this field of asthmatic endophenotype stability, Bobolea et al. have worked to explore the role of periostin and its correlation with high and low T2 phenotypes. This work is of great importance, given that periostin and its correlation with the final phenotypes of asthma may contribute to target T2 cytokine-targeted therapies in a more personalized approach. This thinking raises significant challenges, including the precise identification of patients who would benefit most, the associated costs, and the need for more research to fully understand the long-term impact (7). As we move into the era of personalized medicine, this approach could pave the way toward a more effective and holistic management of a disease that affects millions of people worldwide.

## References

[1] Pérez de Llano L Cisneros C Domínguez-Ortega J Martínez-Moragón E Olaguibel JM Plaza V Response to monoclonal antibodies in asthma: definitions, potential reasons for failure, and therapeutic options for suboptimal response. J Investig Allergol Clin Immunol (2023) 33(1):1–13. 10.18176/jiaci.0857 36040046

[2] Alvarez-Gutiérrez FJ Blanco-Aparicio M Casas-Maldonado F Plaza V González-Barcala FJ Carretero-Gracia JÁ Documento de consenso de asma grave en adultos. Actualización 2022. Open Respir Arch. (2022) 4:100192. 10.1016/j.opresp.2022.100192 37496585 PMC10369632

[3] Guía Española para el Manejo del Asma. GEMA 5.3. (2023). Available online at: www.gemasma.com (accessed December 17, 2023).

[4] Global Initiative for Asthma. Global Strategy for Asthma Management and Prevention. (2023). Available online at: www.ginasthma.com (accessed December 17, 2023).

[5] Couillard S Laugerud A Jabeen M Ramakrishnan S Melhorn J Hinks T Derivation of a prototype asthma attack risk scale centred on blood eosinophils and exhaled nitric oxide. Thorax. (2022) 77(2):199–02. 10.1136/thoraxjnl-2021-217325 34362839 PMC8762000

[6] Casas-Maldonado F Álvarez-Gutiérrez FJ Blanco-Aparicio M Domingo-Ribas C Cisneros-Serrano C Soto-Campos G Monoclonal antibody treatment for severe uncontrolled asthma in Spain: analytical map. J Asthma. (2022) 59(10):1997–07. 10.1080/02770903.2021.1978483 34503370

[7] Buhl R Humbert M Bjermer L Chanez P Heaney LG Pavord I Severe eosinophilic asthma: a roadmap to consensus. Eur Respir J. (2017) 49:1700634. 10.1183/13993003.00634-2017 28461308

